# The Impact of Advanced Image-Guided Breast Surgery and Oncoplastic Techniques on Margin Positivity in Breast Conserving Surgery

**DOI:** 10.7759/cureus.11831

**Published:** 2020-12-01

**Authors:** Tolga Ozmen, Eli Avisar

**Affiliations:** 1 Surgical Oncology, University of Miami, Miller School of Medicine, Miami, USA

**Keywords:** breast cancer, image-guided surgery, resection margins, surgical margins, residual disease, ultrasound

## Abstract

Objective

Positive margins remain a significant psychological and economic burden after breast conserving surgery. The aim of this study was to test the hypothesis that advanced oncoplastic techniques as well as intraoperative integrative imaging with intraoperative ultrasound and mobile digital specimen radiography decreases positive margin rate in breast conserving surgery.

Methods

A single-institution retrospective review of a prospectively collected database was performed. Patients with breast neoplasms who underwent lumpectomy with or without using intraoperative integrative imaging approaches and oncoplastic techniques were included. The primary outcome was positive margin rate for each technique.

Results

A total of 392 patients were included in the study. The median age of the cohort was 59 years. Overall positive margin rate was 15%. Ductal carcinoma in situ (DCIS) histology and larger tumor size were associated with higher positive margin rate. Intraoperative integrative imaging significantly decreased positive margin rate (9% vs. 18%, p=0.018). Oncoplastic techniques also decreased positive margin rate from 16% to 12%, however this was not significant.

Conclusion

Positive margin rate was significantly lower when intraoperative integrative imaging was used. Oncoplastic techniques also decreased positive margin rate in a selected group of patients with large tumor size. We suggest incorporating these techniques in all breast conserving surgery cases.

## Introduction

Breast cancer is the most commonly diagnosed cancer in women with an incidence rate of 13% [1]. The treatment of this common cancer has evolved significantly from radical mastectomy to breast conserving surgery (BCS) [2,3]. BCS offers a significant benefit for many patients by preserving body image, saving breast mound and sensation. These advantages come with a price of increased positive surgical margin requiring additional surgery. Positive margin rate (PMR) is reported as high as 40% in the literature [4-8]. In North America, 25% of patients undergo a re-excision surgery due margin positivity after BCS [9]. Decrease in PMR has become a major research focus leading to major technologic, surgical and research investments. In addition, significant efforts have been made in an attempt to develop consensus and guidelines in the definition and management of positive margin. 

A variety of techniques have been used to localize non-palpable breast cancers including wires, radioactive and non-radioactive seeds, and intra-operative ultrasound (IOUS). Besides localizing the lesion, IOUS can also be used to guide the resection margins in both palpable and non-palpable breast cancers. Some studies claim one technique being superior to another, however the data are conflicting and the gold standard technique is yet to be determined [10-13]. In 2015, the American Society of Breast Surgeons reviewed all the available evidence and developed a toolbox of recommendations to reduce PMR and improve cosmetic outcome after BCS. They concluded that evidence was not definitive to recommend a single technique [14].

For needle localized tumors, the standard approach is to confirm resection of the targeted lesion with specimen radiography. Conventional specimen radiography requires the specimen to be transported to radiology department, while the patient stays in the operating room under anesthesia. Prolonged anesthesia time increases both anesthesia associated morbidity, mortality and the cost associated with operating room occupation [15]. Mobile digital specimen radiography (MDSR), first introduced in 1998, is a mobile X-ray device, allowing specimen images to be obtained in the operating room and interpreted remotely, which bypasses transportation of the specimen. Studies showed that despite lower image quality, MDSR allows verification of target lesion excision in confidence [15-17].

Excising a larger volume of tissue by implementing oncoplastic techniques (OT) also has the potential to decrease PMR without compromising the cosmetic outcome [18]. OT were also included into the American Society of Breast Surgeons’ toolbox of recommendations and the consensus strongly recommended it to be applied for patients with large tumors undergoing BCS [14].

Above mentioned integrated intraoperative imaging (III) techniques and OT are commonly used for patients undergoing breast surgery in our clinic. The aim of this study was to test the effects of III and OT on PMR and to share our experience on margin positivity.

## Materials and methods

This is a retrospective analysis of a prospectively collected database. We queried all women treated in our clinic for invasive ductal cancer (IDC), invasive lobular carcinoma (ILC), ductal carcinoma in situ (DCIS) or Phyllodes tumor between 2010 and 2015. The data included demographics and tumor characteristics as age, histological tumor type, tumor volume and specimen volume as well as surgical data regarding use of OT and/or III and margin positivity. This study was approved by our institutional review board (#20053311) who granted a waiver of consent. The study was compliant with the Health Insurance Portability and Accountability Act (HIPAA). 

During the course of data collection, we experienced a gradual incorporation of MDSR, IOUS, and OT in the operating room among a homogenous and well-trained group of surgeons thus enabling a rare opportunity to compare these techniques with conventional methods in a random, non-tumor and non-patient related fashion.

Definition of terms

Surgeries as flap advancement, breast reduction, mastopexy or adjacent breast tissue advancement were accepted as OT. MDSR and intraoperative IOUS were accepted as III modalities.

During the course of the study, negative margins were defined at our institution as “no tumor on ink” for both invasive and non-invasive cancer. This is in accordance with the subsequently published Society of Surgical Oncology (SSO) and the American Society for Radiation Oncology (ASTRO) consensus guidelines for surgical margins for invasive cancer [19] but slightly more lenient than the later published for DCIS [20]. For Phyllodes tumor, we aimed to excise the tumor with wide local excision (> 10mm). The decision to proceed with re-excision for positive margins was made on a case per case basis and relied on patient and tumor factors as well as a multidisciplinary discussion. 

Statistical analysis

Statistical analysis was carried out using Statistical Package for Social Sciences (SPSS) Statistics version 23 (IBM Corp., Armonk, NY, USA). Student’s T-test was used to compare continuous values, while chi-square test was used for categorical values. A “p” value less than 0.05 was considered statistically significant.

## Results

A total of 392 patients were included in the study. The median age was 59 (27-92) years. PMR was 15%. Invasive ductal carcinoma was the most common histologic tumor type (78%) followed by DCIS (13%), invasive lobular carcinoma (5%) and Phyllodes tumor (4%). OT and III were used in 36% and 32% of the cases, respectively. MDSR was the most commonly used III modality (17%), followed by IOUS (12%). Median excised tumor volume was 3.4 (0.2-1701.1) cm^3^ and median excised specimen volume was 166.5 (1.3-1802.2) cm^3^ (Table 1).

**Table 1:** Descriptive parameters of the cohort Categorical variables are presented as n (%) Continuous variables are presented as Median [Range] åMDSR: mobile digital specimen radiography

Age (y)		59 (27-92)
Positive surgical margin(s)		58 (15)
Oncoplastic technique		142 (36)
Integrated intraoperative imaging	None	265 (68)
	Intraoperative ultrasound	47 (12)
	MDSR^å^	66 (17)
	Both	14 (3)
Histologic type of tumor	Invasive ductal carcinoma	306 (78)
	Ductal carcinoma in-situ	49 (13)
	Invasive lobular carcinoma	19 (5)
	Phyllodes tumor	17 (4)
Volume of tumor (cm^3^)		3.4 (0.2-1701.1)
Volume of specimen (cm^3^)		166.5 (1.3-1802.2)

In our cohort, patients who underwent OT had significantly larger specimen volume (235.2 cm^3^ vs. 188.2 cm^3^, p=0.024). Also, the mean age of patients in the OT group was younger, but this was statistically not significant (56.7 vs. 58.6, p=0.077). III was most commonly used in DCIS patients (44%). A histology of DCIS (31%, p=0.002) and larger tumor volume (51.4 cm^3^ vs. 10.6 cm^3^, p=0.002) were associated with higher margin positivity. III decreased positive margin risk by 56% (9% vs. 18%; p=0.018, odds ratio [OR] 0.44 [0.22-0.88]) (Table 2). 

**Table 2:** Comparison of descriptive parameters with oncoplastic techniques and integrated intraoperative imaging. Categorical variables are presented as n (%) Continuous variables are presented as Mean ±SD

		Oncoplastic Technique		P	Integrated Intraoperative Imaging		P
		No	Yes		No	Yes	
Age (y)		58.6±9.8	56.7±10	0.077	58.1 ±10.3	57.6 ±9.1	0.695
Histologic type of tumor	Invasive ductal carcinoma	189 (62)	117 (38)	0.128	200 (65)	106 (35)	0.032
	Ductal carcinoma in-situ	33 (67)	16 (33)		32 (56)	17 (44)	
	Invasive lobular carcinoma	13 (68)	6 (32)		15 (79)	4 (21)	
	Phyllodes tumor	15 (88)	2 (12)		17 (100)	0 (0)	
Volume of tumor (cm^3^)		16.4±108.5	17±49.4	0.937	19.9 ±110.6	9.8 ±17.1	0.148
Volume of specimen (cm^3^)		188.2±183.7	235.2±204.3	0.024	196.4 ±188.9	223.7 ±199.1	0.199

In multivariate analysis, DCIS histology and larger tumor volume continued to be statistically significant, however, p value for III was 0.052 (Table 3).

**Table 3:** Parameters affecting with margin status Categorical variables are presented as n (%), continuous variables are presented as Mean ±SD *OT = oncoplastic technique; ^w^III = integrated intraoperative imaging ^å^MDSR: mobile digital specimen radiography

		Margin Status		Univariate p	Multivariate p
		Negative	Positive		
Age(y)		57.9±10.1	57.8 ±9.3	0.942	
Histologic type of tumor	Invasive ductal carcinoma	272 (89)	34 (11)	0.002	0.038
	Ductal carcinoma in-situ	34 (69)	15 (31)		
	Invasive lobular carcinoma	15 (79)	4 (21)		
	Phyllodes tumor	12 (71)	5 (29)		
Volume of tumor (cm^3^)		10.6 ±23.9	51.4 ±229.6	0.002	0.015
Volume of specimen (cm^3^)		198.6 ±158.1	243.3 ±326.3	0.103	
OT^*^	No	209 (84)	41 (16)	0.235	
	Yes	125 (88)	17 (12)		
III^w^	No	218 (82)	47 (18)	0.018 OR 0.44 [0.22-0.88]	0.052
	Yes	116 (91)	11 (9)		
III^w^ type	No	218 (82)	47 (18)	0.088	
	MDSR^å^	59 (89)	7 (11)		
	Intraoperative US	43 (91)	4 (9)		
	Both	14 (100)	0 (0)		

In an attempt to better understand the specific contributions of OT and III to the margin status, we analyzed the effect of III on PMR after stratification into OT vs. no OT. In the “no OT” group, III significantly decreased PMR (0% vs. 18%, p=0.026, OR 0.82 [0.77-0.87]). In the “OT” group, III did decrease PMR by 5%, but this difference was not statistically significant (Table 4).

**Table 4:** Effect of III on margin status after stratification by OT Data are presented as n (%), *OT = oncoplastic technique; ^w^III = integrated intraoperative imaging

		Margin Status		p
		Negative	Positive	
No OT^*^	No III^w^	186 (82)	41 (18)	0.026 OR 0.82 [0.77-0.87]
	III^w^	23 (100)	0 (0)	
OT^*^	No III^w^	32 (84)	6 (16)	0.397
	III^w^	93 (89)	11 (11)	

## Discussion

The wide implementation and acceptance of breast cancer screening programs coupled with significant advancements in imaging technology have resulted in a large increase in early breast cancer diagnosis, making BCS the preferred treatment option. The many advantages of breast conservation are beyond the scope of this discussion. BCS, however, is also associated with an inherent significant risk of margin positivity. In the literature, PMR has been reported as high as 40% [4-8]. Positive margins are frustrating to patients and providers alike and are associated with an increase in re-operations, mastectomy rates, medical and radiation treatments all of which are difficult for the patients and expensive for the health care systems. Various techniques have been proposed in an attempt to minimize the PMR with the best approach yet to be determined. In our series, we calculated a PMR of 15%. This rate is slightly lower than the 20-40% PMR range reported in the literature [21, 22]. Previous studies have shown that DCIS histology is associated with an increase in PMR [23-25]. Similarly, in our study, PMR was higher among DCIS patients. Another parameter we found to be associated with a higher margin positivity was larger tumor size. This finding was also previously reported by Singletary et al. [7].

In our study, III decreased PMR risk by 56%. The effect of MDSR on PMR has been previously studied and was shown to decrease margin positivity by 60-96% [25,26]. Versteegden et al. reported in their study that the benefit of radiography was however weakened in in-situ histology, and this was attributed to the extent of disease becoming harder to define in these cases [27]. Karanlik reported an 11% decrease and Krekel reported a 13% decrease in PMR with the use of IOUS for BCS [10,28]. In our study, IOUS decreased PMR to a greater extent than MDSR and the combination of MDSR and IOUS yielded the lowest PMR. We also looked at invasive and in-situ carcinoma separately. In invasive ductal cancer, MDSR alone and IOUS alone decreased PMR similarly (8% and 9% vs. 13% respectively). However, the lowest PMR was achieved when MDSR and IOUS were used together (0% vs. 13%). In in-situ disease, MDSR alone decreased PMR from 38% to 21% and none of the patients underwent IOUS alone in this histology subtype. Once again, the lowest PMR was achieved when MDSR and IOUS were used together (0% vs. 38%). It would appear therefore, that MDSR and IOUS should be used together to achieve the lowest PMR in both invasive and in-situ disease. However, we believe that this should be tested in larger cohorts.

As already mentioned in the introduction, the definition of PMR for in-situ disease was no tumor on ink which is different than the later published recommendation of 2 mm margins. No tumor on ink however, was the accepted definition of PMR in all NSABP studies on in-situ disease and still has a very significant validity. In fact, although the current guidelines recommend a 2 mm margins for in-situ disease, the need for re-excision is left vague as long as no tumor is present on ink. Additional considerations as tumor histology, patient age, additional treatment modalities etc. have to be considered in those cases. Furthermore, the current management of in-situ breast neoplasms continues to be controversial when on one hand wider margins are recommended and on the other hand less surgery and less radiation are been actively studied including observation only. In regard to our series, given the fashion of the data recording and collection as well as the current practice at the time of this study it would have not been feasible to redefine positive margins for in-situ disease. We believe however that the data on PMR as we defined it is still valid and relevant. Potentially, MDSR and IOUS could be similarly used after adoption of wider margins and being associated with similar results, however this will need further studies. 

As the tumor size increases, achieving negative margins along with a successful cosmetic result becomes more challenging. To that end, OT can be of great help to the operating surgeon. In the American Society of Breast Surgeons recommendation toolbox, OT was defined as having the potential to decrease PMR by allowing a larger specimen to be excised [17]. Specimen volume excised in the OT group was significantly larger in our study and indeed the PMR in this group was lower by 4%. The decrease in PMR was more prominent when OT was used in larger tumors suggesting that those cases benefit most from the addition of OT to III. 

Since both OT and III decrease PMR, it is not surprising that in the multivariate analysis III lost some of its efficacy. Its beneficial effect on PMR is however still significant enough so that it barely missed the 0.05 significance. Clearly a larger sample might help demonstrate that strong trend. In the subgroup without use of OT a sharp and statistically significant PMR decrease was easily observed. Different techniques have been described in an attempt to decrease PMR for BCS. One of them, shave cavity margins, have demonstrated a 50% decrease in PMR [7] but the cosmetic results are impacted by removal of additional margins that might not be involved. Additionally, the increase in pathology samples and cost are significant. Other techniques require use of specific instruments meant to identify the positive margins in the operating room. Most of those tools, however, are expensive, add operating time, and are associated with losing specificity in the pursuit of sensitivity. In this study we have demonstrated that PMR can be decreased by roughly 50% and brought down to around 10% by simple use of technology already available and without compromising breast cosmesis.

Some of the limitations of this study include the relatively small sample size and the fact that it is a retrospective analysis. For those reasons further subset analysis is difficult and we cannot correct for possible time variation as, specific surgeons learning curves as well as surgeon-specific factors. It should be mentioned however that the same group of surgeons participated in the whole study and previous QA projects revealed no significant differences in positive margin rates between surgeons. Prospective randomized studies and larger sample size will be necessary to better assess the effects of III and OT on PMR.

## Conclusions

Integrated intraoperative imaging significantly decreases PMR using readily available and inexpensive imaging tools and without additional tissue resection. OT enables better cosmetic results for patients with larger tumor volumes with no compromise of the PMR.
